# Anemia and Blood Transfusions in Critically Ill Patients

**DOI:** 10.1155/2012/629204

**Published:** 2012-10-04

**Authors:** M. Kamran Athar, Nitin Puri, David R. Gerber

**Affiliations:** ^1^Division of Neurocritical Care, Hospital of the University of Pennsylvania, 3 West Gates Building, Philadelphia, PA 19104, USA; ^2^Inova Health System, Falls Church, VA 22042, USA; ^3^Edward D Viner Intensive Care Unit, Cooper University Hospital Camden, Camden, NJ 08103, USA; ^4^Cooper Medical School of Rowan University, Camden, NJ, USA; ^5^Robert Wood Johnson Medical School, University of Medicine and Dentistry of New Jersey, Camden, NJ, USA

## Abstract

Anemia is common in critically ill patients. As a consequence packed red blood cell (PRBC) transfusions are frequent in the critically ill. Over the past two decades a growing body of literature has emerged, linking PRBC transfusion to infections, immunosuppression, organ dysfunction, and a higher mortality rate. However, despite growing evidence that risk of PRBC transfusion outweighs its benefit, significant numbers of critically ill patients still receive PRBC transfusion during their intensive care unit (ICU) stay. In this paper, we summarize the current literature concerning the impact of anemia on outcomes in critically ill patients and the potential complications of PRBC transfusions.

## 1. Introduction

Anemia is a commonly encountered clinical problem in the critically ill [1]. Ninety-five percent of critically ill patients who stay in the intensive care unit (ICU) for 72 hours or greater suffer from anemia and approximately 40% of them receive packed red blood cell (PRBC) transfusions [2, 3]. In 2001, nearly 14 million units of packed red blood cells were transfused, but the physiologic basis for transfusion in the critically ill is not without controversy [4]. In the last two decades transfusion practices have become more restrictive likely in response to prospective research.

## 2. Mechanisms/Etiologies of Anemia in Critically Ill Patients

The etiology of anemia in critical illness is multifactorial and complex. Repeated phlebotomies, gastrointestinal blood loss, and other surgical procedures contribute significantly to the development of anemia [5, 6]. Other factors involved in pathogenesis include coagulopathies, pathogen-associated hemolysis, hypoadrenalism, and nutritional deficiencies [7, 8].

 A number of studies have identified potentially correctible nutritional deficienncies in critically ill patients, including deficiencies of iron, B12, and folate. These deficiencies can lead to ineffective erythropoiesis with resultant anemia [9, 10].

Decreased erythropoietin production and/or impaired bone marrow response to erythropoietin may also play an important role in the development of anemia [11]. These effects are mediated by a variety of inflammatory cytokines such as Interleukin-1 (IL-1) and tumor necrosis factor-*α* (TNF-*α*), which inhibit erythropoietin (EPO) production. Furthermore IL-1, IL-6, and TNF-*α* suppress erythropoiesis by direct inhibitory effects on bone marrow [12]. 

The hyperadrenergic state following severe injury may also induce bone marrow dysfunction and failure of erythropoiesis. This effect may be mediated by IL-6 and interferon-*γ* (IFN-*γ*), which are released following severe injury and have been shown to inhibit the differentiation and proliferation of erythroid precursor cells [13]. 

In addition increased levels of hepcidin, an iron regulatory hormone, have been purported to play an important role in the development of anemia of chronic disease (ACD) [14–16]. The most common causes of ACD are acute or chronic inflammatory conditions such as infections, cancer, autoimmune diseases, and chronic kidney disease (CKD) [17]. It is characterized by sequestration of iron in macrophages, hypoferremia, and iron-restricted erythropoiesis. ACD is associated with elevated levels of inflammatory cytokines, including IL-1, IL-6, and IFN-*γ* [18]. These cytokines induce excess hepcidin production, which has been shown to downregulate ferroportin, an iron export protein on the cell surface of duodenal enterocytes, macrophages, and hepatocytes [19, 20]. Thus high serum levels of hepcidin decrease intestinal iron absorption and block iron export from tissue stores, resulting in functional iron deficiency [14, 21, 22].

## 3. Impact of Anemia on Outcomes in Critically Ill Patients

### 3.1. Anemia and Cardiac Disease

Numerous recent studies have shown anemia to be associated with worse outcomes in patients with coronary artery disease. After reviewing the data on nearly 40,000 patients enrolled in trials on acute coronary syndrome (ACS) Sabatine et al. found anemia to be associated with a greater likelihood of death in patients with ST-segment elevation MI (STEMI) and non-ST-segment elevation MI (NSTEMI) [23]. These investigators also found an increased association of anemia with recurrent ischemia or acute MI in patients with NSTEMI. Aronson et al. found that lower nadir hemoglobins in hospitalized patients following MI were strongly associated with increased mortality [24].

Kulier et al., in a study of 5065 patients who underwent coronary artery bypass grafting (CABG), found preoperative anemia and intraoperative blood transfusion to both independently be associated with adverse postoperative neurologic and renal outcomes [25]. In another study of over 6,000 patients undergoing percutaneous intervention, anemia was found to be independently associated with both short-term cardiovascular events and decreased 1-year survival [26]. 

### 3.2. Anemia and Respiratory Failure

Respiratory dysfunction has also been associated with anemia in the critically ill. 

Rady and Ryan in a retrospective study of cardiac surgery patients found anemia (defined as a hematocrit <34%) and massive transfusion (defined as >10 units of blood products) to be significantly associated with the need for reintubation [27]. 

Khamiees et al. in a prospective observational study found anemia (Hgb < 10 g/dL) to be associated with extubation failure in a mixed medical-surgical ICU population [28]. Nevins and Epstein found anemia (mean hematocrit 36) to be associated with poor outcomes in a retrospective study of 166 patients with chronic obstructive pulmonary disease (COPD) receiving mechanical ventilation [29]. Although, data exists showing anemia to be associated with poorer outcomes in mechanically ventilated patients, no significant literature supports the transfusion of PRBC to facilitate weaning patients from mechanical ventilation [30].

### 3.3. Anemia and Sepsis

Anemia is also a common occurrence in the setting of sepsis [31]. This is in part because mediators of sepsis (e.g., TNF-*α* and IL-1*β*) decrease expression of the erythropoietin gene and protein [32]. The early-goal-directed therapy trial reported by Rivers et al. and the transfusion requirement in critical care Trial (TRICC) performed by Hebert et al. used two different transfusion strategies in critically ill patients [31, 33]. Rivers et al. showed that correcting anemia by transfusing to a hematocrit >30% in early sepsis was associated with improved outcome when included as part of a broader resuscitation regimen [33]. However, it must be remembered that this was one of a number of interventions as part of an early goal directed protocol. It is unclear how much of the improvement in outcome can be attributed to transfusion alone. In the TRICC trial, subgroup analyses of older or more severely ill patients showed no difference in 30-day mortality between the restrictive-strategy and liberal strategy groups. Younger and less ill patients had better outcomes if transfused in a more restrictive manner (i.e., at lower hemoglobin) [31]. 

## 4. Indications for Transfusion

### 4.1. Transfusion and Tissue Hypoxia

The goal of packed red blood cell transfusion in the critically ill is to increase oxygen delivery to and hence consumption by tissues. An increase in hemoglobin should improve patient's oxygen carrying capacity and help deliver oxygen to hypoxic tissues. Lactate, a clinically used surrogate of tissue hypoxia, should decrease with improved oxygen consumption. An abundance of literature over the past thirty years demonstrates that this clinical benefit is frequently not realized with transfusion in the critically ill. Shah et al. described their experience with 8 trauma patients who were transfused to a hemoglobin of 10 and had increased oxygen delivery but no increase in oxygen consumption [34]. Dietrich et al. in a larger study in the early nineties transfused volume resuscitated critically ill patients to a hemoglobin of 10 g/dL [35]. These patients again had increased calculated oxygen delivery but no increased oxygen uptake, no decrease in lactate, and no increase in cardiac index. 

Silverman and Tuma used intramucosal gastric pH, a marker for adequacy of tissue oxygenation, to demonstrate that transfusion was inferior to dobutamine in improving oxygen delivery to the splanchnic circulation [36]. Marik and sibbald evaluated 23 critically ill patients with sepsis in a prospective study and demonstrated that transfused patients had no increase in oxygen uptake [37]. Furthermore, they demonstrated that blood older than 15 days decreased patient's gastric intramucosal pH, suggesting an impairment in systemic oxygen delivery at the cellular level. Mazza et al. in a prospective study in 2005 showed that transfusing patients to a hemoglobin above 9 g/dL was not associated with a significantly decreased lactate or increased mixed venous oxygen saturation, indication no benefit in meeting previously unmet tissue oxygen needs [38]. These studies raise the question: why does the transfusion of packed red blood cells frequently not relieve tissue hypoxia? 

The mean age of blood transfused in the USA is 17.9 days and extensive literature exists about age-related changes of packed red blood cells [2]. Stored blood cells have decreased amounts of 2,3-diphosphoglycerate which shifts the oxygen disassociation curve to the left, decreasing hemoglobin's ability to unload oxygen at the tissue level.Stored erythrocytes have been shown to lose their deformability resulting in increased aggregation, hemolysis, and the subsequent release of systemic factors associated with organ dysfunction [39, 40]. Fitzgerald et al. demonstrated in rats that transfused blood which was less than 3 days old was associated with an increased systemic oxygen uptake as opposed to blood that was 28 days old [41]. Zallen et al. demonstrated that an increased mean age of blood, especially an age of stored blood greater than 2 weeks, was associated with multiple organ failure [42].

## 5. Complications of Transfusion

### 5.1. Infection

Historically, the AIDS crisis forced the critical care community to revaluate the benefits of blood transfusion in the ICU. Due to these concerns blood is now screened for a variety of viral and bacterial infections including HIV and Hepatitis A, B, and C. In the United Kingdom, the risk of acquiring infection due HIV secondary to transfusion is less than one in 2 million [43]. 

The transmission of infection is only one of the ways blood can affect its potential recipient. In the early seventies, it was observed that renal transplant patients who received a transfusion prior to transplant had increased allograft survival. This finding led to further postulation in the medical literature that the transfusion of blood might affect the recipient's immune system. Over the past forty years numerous studies have evaluated the affect of transfusing on transplant survival, cancer recurrence, and host predisposition to infection [44]. 

Taylor et al., in a retrospective study of 1711 patients demonstrated that transfused patients were six times more likely to develop a nosocomial infection than nontransfused patients [45]. In addition, each unit of PRBC transfused increased patients odds of developing infection by 1.5. Claridge et al. demonstrated in a prospective study of 1593 trauma patients that 33.6% of transfused patients developed infection versus 7.6% patients who did not receive transfusions [46]. They also showed that a linear, dose-response pattern existed, as patients who received more PRBC were more likely to develop infection. Univariate analysis of their data showed that the strongest predictor of developing infection was the transfusion of PRBC in the first 48 hours. Shorr et al. conducted an important secondary analysis of the CRIT study involving 284 ICUs and 4,892 patients to evaluate for blood stream infections (BSIS) in the critically ill [47]. These investigators demonstrated that three factors were associated with new BSI in the ICU: the initial use of cephalosporins, higher sequential organ failure assessment score, and PRBC transfusion. The importance of their work is that the data came from multiple ICUs unlike single-center studies mentioned previous and the patient populations were heterogeneous. Shorr et al. did another secondary analysis of the CRIT study and demonstrated that both small (1-2 units) and large (>2 units) volume transfusions were independent risk factors for the development of ventilator-associated pneumonia [48]. In cardiac surgery patients the transfusion of PRBC has been similarly associated with increased risk of infection [30]. However, Ali et al. in a prospective, single-center study of 234 patients demonstrated that PRBC was not associated with increased risk of infection in postoperative cardiac surgery patients [49]. Multiple large well-designed trials suggest that PRBC predispose patients to infection likely to due the immunomodulation of the patient's immune system, but as Ali et al. study demonstrates that debate in this area still exists. 

### 5.2. Transfusion-Related Acute Lung Injury (TRALI)

This is a clinical syndrome that presents as acute hypoxemia and noncardiogenic pulmonary edema generally occurring within six hours of a transfusion [50]. The incidence varies and is estimated at 1 in 5,000 blood and blood components [51]. According to an FDA report presented at the TRALI conference in 2004, it is now the leading cause of transfusion-related death in the United States. TRALI is characterized by increased pulmonary microvascular permeability. Leukocyte antibodies and biologically active substances such as lipids and cytokines are thought to be responsible for this increased permeability [52, 53]. 

## 6. Impact of Blood Transfusion on Clinical Outcomes

### 6.1. Large Studies about Transfusion Outcome

A growing body of evidence now suggests that correcting anemia by transfusion often either provides no benefit or is harmful. The TRICC trial showed a significant increase in cardiac and pulmonary complications and a trend toward increased mortality in the liberal transfusion group during patients intensive care stay. In subgroup analysis, younger (age < 55 yrs) or less critically ill (APACHE II scores < 20) patients randomized to a liberal strategy had a statistically significant increase in mortality [31]. 

The CRIT study found that the number of blood transfusions was independently associated with both length of stay and mortality in ICU patients [2]. A cohort analysis within the CRIT study found blood transfusion to be independently associated with development of acute respiratory distress syndrome (ARDS) [54]. 

In 2002, Vincent et al. published a prospective observational study (Anemia and Blood Transfusion in Critically Ill Patients ABC) evaluating the blood sampling, hemoglobin levels, and transfusion rates in 146 Western European ICUs [3]. They concluded that receipt of a blood transfusion increased a patients odds of dying and increased patients length of stay in the ICU. In 2008, Vincent el al. published another prospective observational study that included 198 European ICUs [55]. This study showed that transfused patients had a lower mortality than nontransfused patients at 30 days when patient populations were matched by propensity scores. Significant debate exists about the use of a propensity score for statistical analysis including the inability to balance unmeasured clinical variables in the studied populations [56]. Vincent et al. have spurred further debate about the need for another prospective trial comparing a liberal versus conservative blood transfusion strategy in the critically ill, especially in the era of leukodepletion of PRBC transfusions. 

### 6.2. Transfusion Impact on Cardiac Disease

Data from the initial TRICC trial demonstrated that patient in the liberal transfusion arm (i.e., those transfused at a threshold hemoglobin of 9 g/dL versus 7 g/dL for the restrictive group) had a higher incidence of both pulmonary edema and MI [31].Subsequently, a subgroup analysis of patients from the TRICC trial with heart disease failed to demonstrate any significant mortality outcomes between groups. However, patients in the liberal transfusion group had a greater incidence of organ dysfunction [57]. 

Wu et al. reviewed Medicare data on almost 79,000 patients aged >65 years admitted with a diagnosis of acute MI [58]. They found an association between blood transfusion and improved survival when admission hematocrit was <33%. Any benefit of transfusion was lost at a hematocrit >33%, and patients with hematocrit >36% who were transfused had an increased risk of death compared with nontransfused patients. 

Yang et al. found that blood transfusion was a risk factor for death and the combined end point of death or MI in patients with non-ST-elevation ACS [59]. 

Rao et al. found a similar association with blood transfusion for patients with ACS for hematocrits more than 25% [60]. Below this value there was no difference in mortality between transfused and nontransfused patients.

Sabatine et al. [23] found differing results for patients with STEMI and those with non-ST-elevation ACS. Patients with STEMI and hemoglobin levels <12 g/dL had improved outcomes when transfused. Conversely, patients with non-ST-elevation ACS were found to have worse outcomes if transfused, regardless of their hemoglobin level.

Numerous studies in cardiac surgery patients have identified blood transfusion to be independently associated with increase in infectious complications, myocardial infarction (MI), stroke, renal failure, prolonged mechanical ventilation, atrial fibrillation, hospital length of stay, and mortality [61–63].

### 6.3. Blood Conservation

Patients in the ICU on average have 41ml of blood drawn over a 24-hour period with sicker patients requiring more frequent blood draws leading to even greater blood loss [3]. Older literature has estimated that patients with an arterial catheter could lose greater than 900 mL during their stay [64]. Educational programs about reduced sampling, point of care testing, and closed arterial line system have all been recommended [3].

### 6.4. Alternatives to Transfusion

Ideally, mechanisms would exist to decrease or eliminate the need of blood transfusion altogether. Critically ill patients with anemia have been shown to have an inappropriately low erythropoietin response to their disease [65]. Corwin et al. in two studies demonstrated that transfusion requirements were decreased in critically patients treated with erythropoietin. However, in their third study erythropoietin treatment did not significantly alter the rate of transfusion in the critically ill. Mortality was nonsignificantly different in the medical population, but significantly lower in trauma patients at 140 days, although this decrease in mortality was unrelated to any difference in transfusion rates in the groups that received and did not receive erythropoietin. This observation led the authors to hypothesize that erythropoietin decreases mortality in trauma patients by mechanisms other than the reduction in PRBC requirements [66]. Erythropoietin is not recommended in critically ill medical patients unless other medical indications exist and can be used in trauma patients who will be in the ICU for greater than 48 hours. 

Another mechanism for reducing PRBC transfusion is artificial oxygen carriers or hemoglobin substitutes. Hemoglobin substitutes can be divided into two categories, perfluorocarbons and modified hemoglobins. Perfluorocarbons transport both oxygen and carbon dioxide but require patients to be on 100% oxygen. The advantages of perfluorocarbons over PRBC are their lack of potential infection transmission and their long half-lives. They have been shown to decrease the need for blood transfusions in patients with elective noncardiac surgeries. Further studies need to be done to determine their utility in the critically ill [67]. 

Although, bovine, old human, and recombinant-based hemoglobin-based oxygen carriers (HBOC) have been heavily researched for thirty years only one HBOC is approved for clinical use. Hemopure (Biopure http://www.biopure.com/) was approved in 2001 to help reduce the amount of blood transfusion in anemic surgical patients in South Africa. The utility of these hemoglobin-based blood products currently is thought to be the ability to decrease allogenic PRBC transfusion and provide a blood substitute in situations where blood is unavailable (combat). Significant debate exists as to whether there are more myocardial infarctions in patients who receive HBOC as one meta-analysis suggested [68]. The National Heart, Lung, and Blood Institute in 2006 convened a conference to examine the difficulties with the development of HBOCS. The results of the conference were that HBOC show potential to have clinical utility, but concerns over side effects have impeded their approval by regulatory agencies [69].
